# The B subunit of *Escherichia coli* enterotoxin helps control the in vivo growth of solid tumors expressing the Epstein–Barr virus latent membrane protein 2A

**DOI:** 10.1002/cam4.380

**Published:** 2015-02-02

**Authors:** Beatrice Ondondo, Lee Faulkner, Neil A Williams, Andrew J Morgan, David J Morgan

**Affiliations:** 1 The Jenner InstituteOld Road Campus Research Building, Roosevelt Drive, Oxford, Oxfordshire OX3 7DQ, United Kingdom; 2 Institute of Translational Medicine, University of LiverpoolCrown Street, Liverpool, L69 3BX, United Kingdom; 3 School of Cellular and Molecular Medicine, University WalkMedical Sciences Building, Bristol, BS8 1TD, United Kingdom

**Keywords:** Adjuvant, cytotoxic T-lymphocyte, Epstein–Barr virus, murine

## Abstract

Latent membrane protein 2A (LMP2A) is expressed on almost all Epstein–Barr virus (EBV)-associated tumors and is a potential target for immunotherapeutic intervention and vaccination. However, LMP2A is not efficiently processed and presented on major histocompatibility antigens class I molecules to generate potent cytotoxic T-lymphocytes (CTL) responses capable of killing these tumors. The B subunit of *Escherichia coli* enterotoxin (EtxB), causes rapid internalization and processing of membrane-bound LMP2A on EBV-infected B cells, and facilitates loading of processed-LMP2A peptides onto MHC class I. This re-directed trafficking/delivery of LMP2A to the MHC class I machinery enhances recognition and killing by LMP2A-specific CTL in vitro. To test the potential of EtxB to enhance immune targeting of LMP2A expressed in solid tumors, we generated a murine tumor model (Renca-LMP2A), in which LMP2A is expressed as a transgenic neoantigen on a renal carcinoma (Renca) cell line and forms solid tumors when injected subcutaneously into BALB/c mice. The data show that in BALB/c mice which have only low levels of peripheral K^d^-LMP2A-specific CD8^+^ T cells, merely a transient inhibition of tumor growth is achieved compared with naïve mice; suggesting that there is suboptimal LMP2A-specifc CTL recognition and poorly targeted tumor killing. However, importantly, treatment of these mice with EtxB led to a significant delay in the onset of tumor growth and significantly lower tumor volumes compared with similar mice that did not receive EtxB. Moreover, this remarkable effect of EtxB was achieved despite progressive reduction in tumor expression of LMP2A and MHC class I molecules. These data clearly demonstrate the potential efficacy of EtxB as a novel therapeutic agent that could render EBV-associated tumors susceptible to immune control.

## Introduction

The key to generating effective anti-cancer vaccines is the ability to stimulate cytotoxic T-lymphocytes (CTL) responses against tumor antigens 1,2. For virus-associated cancers, CTL targeting of the viral antigens could potentially control tumor growth. For example Epstein–Barr virus (EBV)-associated malignancies such as nasopharyngeal carcinoma (NPC) and gastric cancer (GC) express latent membrane protein 2A (LMP2A) which is a suitable CTL target 3,4. However, effective ways of delivering LMP2A in order to activate potent antitumor CTL responses still remain a major challenge. Recent studies have shown that potent clinical responses may be achieved in certain patients with EBV-associated lymphomas by administering LMP2A-specific T cells or by vaccinating with LMP2A-transduced DC 5. However, despite such successes, there will be limitations with these types of approaches in tackling other EBV-associated tumors that maybe much less immunogenic; undergoing immunoediting to escape detection. Therefore, it is likely that the use of potent adjuvants during such immunotherapeutic approaches ought to enhance their efficacy.

The B subunit of *Escherichia coli* enterotoxin (EtxB) has already been used as a vaccine adjuvant, and was shown to enhance immune responses to various coadministered antigens in vivo 6–8. Its adjuvant properties are linked to the ability to bind the cell surface receptor ganglioside GM_1_ 9 found in lipid rafts of mammalian cells 10. Chemical conjugation of peptides and/or short proteins to EtxB has also been demonstrated to facilitate their external delivery into the MHC class I cross-presentation pathway, thus enhancing CD8^+^ T-cell responses to poorly presented antigens 11–13. Previous studies showed that EtxB is also able to enhance antigen processing and presentation via the endogenous MHC class I pathway 14.

Studies which examined the effect of EtxB upon EBV class I antigen processing and presentation clearly demonstrated that EBV-infected lymphoblastoid cell lines (LCL) that had been exposed to EtxB were killed much more efficiently by CTL specific for the EBV LMP1 and LMP2. These proteins are colocalized in the GM_1_-rich, lipid microdomains in the plasma membrane of lymphocytes 15,16 and have a number of roles in EBV latency and cell growth control 17,18. LMP2 is found in two isoforms, LMP2A and 2B, the latter not carrying the N-terminal 119 amino-acid cytoplasmic domain 19. However, when expressed by epithelial tumor cells these proteins are found on internal cell membranes in a less ordered state, and on the cell surface 20.

The colocalization of EtxB with LMP1 and LMP2A in the lipid rafts of LCLs is thought to result in redirected trafficking of these proteins during the cross-aggregation and rapid internalization of EtxB following GM_1_ binding. As EtxB is transported to the endoplasmic reticulum by retrograde mechanisms 21, it possibly alters the processing pathway of LMP1 and LMP2 leading to enhanced recognition and CTL targeting. Importantly, our previous studies also indicate that an oral epithelial tumor cell line (H103) transfected with EBV LMP2A was not susceptible to killing by HLA-matched LMP2A-specific CTLs unless the target cells were pre or cotreated with EtxB (AJM unpublished data).

A number of human tumors such as NPC, posttransplant lymphoproliferative disease (PTLD), a proportion of gastric carcinomas and Hodgkin's disease (HD) are associated with EBV. These tumors all express the viral nuclear antigens EBNA1 and LMP2 along with LMP1 in varying degrees 22–24. Recognition of EBNA1 by CD8+ T cells is limited in vitro by a glycine–alanine repeat that blocks proteasomal degradation 25,26 and LMP1-specific responses are extremely rare for reasons unknown 27. Therefore, LMP2A stands out as an important virus-associated tumor antigen for immunotherapeutic targeting, and as such, a number of LMP2A CTL epitopes have been mapped 3,28.

Yet, simply immunizing individuals with one of these epitopes in order to generate CTL responses may not be an effective means of controlling tumors. We hypothesized that EtxB could modulate the processing and presentation of LMP2A expressed on EBV-associated solid tumors; thus enhancing their susceptibility to LMP2A-specific CTL, which in turn could prevent the establishment of tumors, delay their onset, reduce the growth rate of emerging tumors, or even cause regression of already established tumors.

Animal models for infection by EBV or closely related animal viruses are currently either inappropriate or experimentally inaccessible. These include the “humanized” mouse 4, the SCID mouse 29,30, murine herpes virus-68 in mice 31, rhesus macaques 32, nude mice, 33 and cotton-top tamarins 34. This has hindered the development of therapies and vaccines targeting LMP2A. In order to overcome this, we have generated a novel LMP2A-expressing tumor model (Renca-LMP2A) which is based on the murine renal carcinoma cell line 35,36. We show that Renca-LMP2A cells form solid tumors that express LMP2A in syngeneic BALB/c, mice and that these tumors can be recognized and targeted by LMP2A-specific T cells; although this is not sufficient to control tumor progression. Most importantly, we demonstrate that administration of EtxB to Renca-LMP2A tumors not only significantly delayed the onset of tumor growth, but moreover caused regression of some tumors; thus demonstrating the strong immunomodulatory potential of EtxB in enhancing antitumor immunity in EBV-associated cancers.

## Materials and Methods

### Mice

Six- to 8-week-old female BALB/c (H-2^d^) mice were purchased from Harlan or B&K and housed under specific pathogen-free conditions within the University of Bristol Animal Services Unit. Experiments were conducted in compliance with UK Home Office regulations.

### Recombinant vaccinia viruses and EtxB

Vac-LMP2A, a recombinant Vaccinia virus expressing EBV LMP2A was constructed as previously described 37, and propagated in 143B or B2-1 cells. Vac-WR (Western Reserve) was used as a control. Recombinant EtxB, prepared as previously described 6, had greater than 98% purity and ≤0.06 of LPS endotoxin unit (EU)/*μ*g, as assessed by the *Limulus* amebocyte lysate assay.

### Construction of a recombinant vector expressing EBV-LMP2A

The EBV LMP2A gene was cloned in pCMVcyto vector (Invitrogen Life Technologies, Paisley, UK) and the resulting pCMVcyto-LMP2A construct used to transform *E. coli* cells. Plasmid DNA was prepared and the size and sequence of LMP2A confirmed by restriction enzyme analysis, PCR amplification, and sequencing of the PCR fragment and the miniprep DNA.

### Tumor cell lines

The renal carcinoma cell line (Renca-WT) 36 was maintained in complete medium (Roswell Park Memorial Institute 1640, 10% FCS, 2 mmol/L glutamine, 50 units/mL penicillin/streptomycin, and 5 × 10^−5^ mol/L 2-mercaptoethanol). Renca-VC and Renca-LMP2A were obtained by transfecting Renca-WT with the empty pCMVcyto or the recombinant pCMVcyto-LMP2A vector, respectively, by the Lipofectamine method (Invitrogen Life Technologies). These cell lines were maintained in complete medium with 0.5mg/mL geneticin (Invitrogen).

### Vaccinia immunization, tumor injection, and EtxB administration

For tumor induction 1 × 10^6^ tumor cells (i.e., Renca-WT, Renca-VC and Renca-LMP2A) in a volume of 100 *μ*L of Phosphate Buffered Saline was injected subcutaneously (s.c.) at the scruff of the neck. Vac-LMP2A or Vac-WR were administered by intraperitoneal (i.p.) injections of 2 × 10^5^ or 2 × 10^6^ plaque forming units (pfu) in a volume of 200 *μ*L. EtxB was delivered in a single injection of 50 *μ*g in 100 *μ*L of PBS at the tumor site.

### LMP2A peptides

H-2K^d^-restricted EBV-LMP2A peptide; LMP2A [AAALALLASLIL_305-316_], a kind gift from (Prof. Zeng Yi, Chinese Center for Disease Control, Beijing, was synthesized by United Peptide Corporation [Bethesda, USA]), at a purity of >91%. The peptide was initially diluted in DMSO at 20 mg/mL and then further diluted in PBS and was used at a final concentration of 10 *μ*g/mL in tissue culture assays.

### Lymphocyte isolation

Tumors were excised and teased apart in serum-free medium using forceps. The cell suspension was passed through a 70-*μ*m nylon cell strainer (BD falcon, Franklin Lakes, NJ), then separated over NycoPrep (Axis Shield, Dundee, UK), and cells at the Ficoll interphase were collected. Spleens were disrupted by passing through a 40-*μ*m nylon cell strainer (BD falcon) using a sterile 2 mL syringe plunger. The cell suspension was centrifuged, and red blood cells lysed using ACK lysis buffer (BD Biosciences, Oxford, UK).

### Antibodies and flow cytometry

Cells were first stained with fluorochrome-conjugated monoclonal antibodies (mAb) against cell surface markers. Fc receptors were blocked using supernatant from anti-FcgIIIR mAb-secreting 2.4G2 cell line and intracellular IFN-*γ* or IL-2 detected using a Cytofix/Cytoperm Plus kit with GolgiPlug (BD) according to the manufacturer's instructions. In assays detecting CD107a, this antibody was added to the cells/medium at the start of stimulation. Stained cells were acquired on a BD LSRII flow cytometer and analyzed with FACSDIVA software. The antibodies used were anti-IFN-*γ*-APC (BD), anti-IL2-PE (BD), anti-TCR*β*-APC-eFluor-780 (eBiosciences, Hatfield, UK), anti-CD4-PerCP-Cy5.5 (BD), anti-CD107a-PE (BD), and anti-CD8-FITC (BD). Expression of MHC class I molecules on tumor cells was quantified by staining with FITC-conjugated anti-H2-D^d^ (BD Pharmingen) and PE-conjugated anti-H-2K^d^ (BD Pharmingen, Oxford, UK). To quantify LMP2A expression by tumor cells, the cells were first fixed with 4% v/v paraformaldehyde, permeabilized with 0.1% wt/v saponin solution in PBS and stained with 1:100 dilution of a rat monoclonal antibody to LMP2A (14B7), followed by a 1:100 dilution of PE-conjugated goat anti-rat IgG (Invitrogen). Cells stained with secondary antibody alone were used as controls.

### LMP2A detection by confocal immunofluorescence

Vac-LMP2A infected B2-1 or 143B cells and Renca-LMP2A tumor cells were fixed with 4% v/v paraformaldehyde, permeabilized with 0.1% v/v saponin solution and stained with 1:50 dilution of a rat mAb to LMP2A (14B7), followed with 1:50 dilution of FITC-conjugated goat anti-rat IgG (Invitrogen Life Technologies, Paisley, UK). Cells were visualized using 63× oil immersion lens on a confocal immunofluorescence microscope and images captured with Leica Confocal Software (Leica, Milton Keynes, UK).

### Generation and maintenance of in vitro T-cell lines

Cells from spleens, lymph nodes, or tumors were used as responder cells to establish T-cell cultures. Renca-LMP2A T-cell lines were established in 24-well plates using 2 × 10^6^ responder cells and irradiated Renca-LMP2A cells as stimulators. These were used at a responder to stimulator ratio of 10:1. The cell cultures were restimulated every 7 days with irradiated Renca-LMP2A and irradiated splenocytes as feeders. From week 2 of culture, cells were grown in complete RPMI medium supplemented with 2% wt/v Concanavalin-A (Con-A) supernatant 38. After at least three rounds of restimulation the cultures were analyzed for evidence of antigen-specific T-cell activity. LMP2A-peptide T-cell lines were set up using irradiated peptide-pulsed splenocytes. Pulsing was performed by incubating the cells for 2 hours at 37°C with 10 *μ*g/mL of peptide after which the peptide washed off. These cultures were also maintained by weekly restimulation with irradiated peptide-pulsed splenocytes.

### Carboxyfluorescein diacetate succinimidyl ester proliferation assay

Single-cell suspensions were labeled with carboxyfluorescein succinimidyl ester (CFSE) as per the manufacturer's guidelines (Invitrogen Life Technologies, Paisley, UK). 1 × 10^5^ CFSE-labeled cells were plated in 96-well round bottom plates in a volume of 100 *μ*L of complete medium and cultured for 4 days either alone or with irradiated peptide-pulsed splenocytes. Plate-bound anti-CD3 (10 *μ*g/mL; eBiosciences mAb) and soluble anti-CD28 (10 *μ*g/mL; eBiosciences) were used as positive controls. After 4 days the cells were stained for surface markers and analyzed by flow cytometry.

### Tumor measurement

Calipers were used to measure the longitudinal diameter (parameter A) and transverse diameter (parameter B) of tumors at specific time points. Tumor volume (in mm^3^) was calculated using the formula; A^2^ × B/2 35.

### Statistical analysis

Statistical analyses were performed using GraphPad prism (version 3.02). Mann–Whitney *U*-test (two-tailed) was used to compare median, whereas two-tailed unpaired *t*-test with Welch's correction was used compare mean tumor volumes. One-way analysis of variance (ANOVA) was used to analyze tumor growth rates, whereas Logrank test was used to compare time to maximum allowed tumor size (MATS). A *P-*value of 0.05 was considered significant.

## Results

### Generation and characterization of an LMP2A-expressing epithelial carcinoma cell line (Renca-LMP2A)

**Figure 1:**
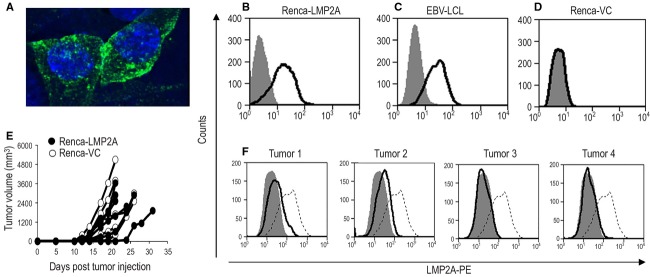
In vitro and in vivo characterization of Renca-LMP2A. The stably transfected Renca-LMP2A cell line was tested for LMP2A expression by (A) Confocal immunofluorescence staining showing LMP2A in green and DAPI staining of the nucleus in blue. (B–D) Show flow cytometric analyses, using PE-labeled LMP2A-specific mAbs, of (B) the Renca LMP2A cell line, (C) EBV-transformed LCLs, and (D) the Renca-vector only control (VC) cell line. For the in vivo characterization, BALB/c mice were injected with either Renca-VC or Renca-LMP2A cells, and tumor measurements recorded for 32 days. (E) Shows the growth profiles of tumors in BALB/c mice injected with Renca-LMP2A (filled circles), and Renca-VC (open circles). (F) Tumors from four of the mice that were given RencaLMP2A were harvested on day 32 and the level of LMP2A expression ex vivo among disassociated Renca-LMP2A tumor cells (black line) versus in vitro-cultured Renca-LMP2A cells (dotted line) was determined by flow cytometric analyses using PE-labeled anti-LMP2A mAbs. The filled histograms represent isotype controls.

Complementary DNA (cDNA) encoding the EBV (B95.8) LMP2A gene was cloned into the pCMV/cyto vector and the recombinant pCMV/cyto-LMP2A DNA used to transfect Renca-WT 33,34 cells, in order to generate a Renca line expressing LMP2A (Renca-LMP2A). Transient and stable expression of LMP2A by the Renca-LMP2A cell line in vitro could be demonstrated by confocal immunofluorescence (Fig.1A) and flow cytometry (Fig.1B). The confocal images show expression of LMP2A in a punctate manner within the cell, excluding the nucleus. LMP2A expression was comparable to that found among control EBV-transformed LCL (Fig.1C). Wild type (WT) Renca cells were also transfected with the vector alone creating the Renca-VC cell line that served as a negative control for Renca-LMP2A cells. As expected, LMP2A expression was not detected on Renca-VC (Fig.1D), but successful transfection was demonstrated by the ability of Renca-VC to grow in selection medium containing geneticin, indicating the presence of the *neomycin* resistance gene found on the vector. Both Renca-LMP2A and Renca-VC grew at a similar rate to each other in vitro, indicating that expression of LMP2A did not alter or affect the fitness of transfected cells in vitro.

### In vivo growth and characterization of Renca-LMP2A tumors

Renca-VC and Renca-LMP2A cells were injected s.c. into BALB/c mice in order to characterize their growth kinetics and in vivo expression of LMP2A and MHC class I molecules. Tumors developed at around day 11 following injection and reached the MATS, according to UK Home Office guidelines, within 3–5 weeks. As shown in Figure1E, there was no difference in the growth profiles of Renca-VC and Renca-LMP2A tumors, and most importantly both profiles were similar to that of Renca-WT tumors (data not shown) in terms of average time to tumor onset and progression to MATS. Subsequent in vivo experiments were thus performed with only Renca-VC as a control for Renca-LMP2A because both of these cell lines were transfected with the pCMVcyto vector.

To test the in vivo expression of LMP2A and MHC class I molecules, tumors from four mice inoculated with Renca-LMP2A were excised and disrupted to form single-cell suspensions. Flow cytometry analyses of LMP2A expression levels by these tumors revealed that, when compared with in vitro*-*cultured Renca-LMP2A, all of the excised tumor cells had reduced levels of LMP2A expression (Fig.1F), with tumors 3 and 4 having virtually undetectable levels of LMP2A compared with in vitro-cultured Renca-LMP2A. However, when these tumor cells were cultured in vitro in the absence of geneticin for 2 weeks, LMP2A expression was restored (Fig.1G).

**Figure 2:**
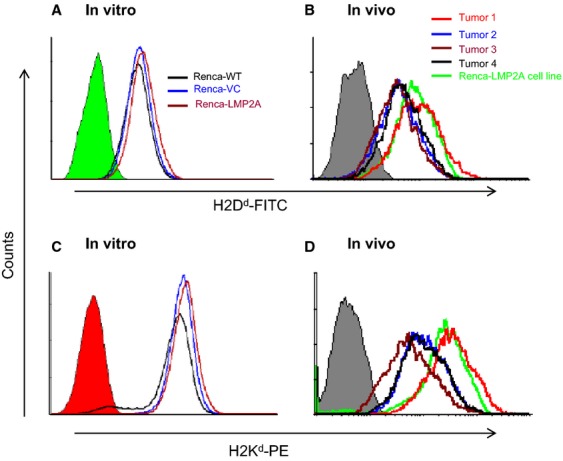
Flow-cytometric analyses of MHC class I H-2D^d^ and H-2K^d^ expression by and Renca-LMP2A cells from in vitro and in vivo culture. In vitro-cultured Renca-WT, Renca-VC and Renca-LMP2A cell lines and Renca-LMP2A cells disassociated from 21 day old solid tumors grown in four separate BALB/c mice were stained with mAbs against H-2D^d^ H-2K^d^. Top histograms show H-2D^d^ expression and Bottom histograms show H-2K^d^ expression by: (A and C) in vitro-cultured cell lines as shown, and (B and D) freshly harvested disassociated Renca-LMP2A tumor cells. The filled histograms represent isotype controls.

Transfection of Renca-WT cells with either the vector alone or the recombinant vector expressing LMP2A did not affect expression of MHC class I molecules in vitro as evidenced by similar expression levels of H-2D^d^ and H-2K^d^ by Renca-WT, Renca-VC, and Renca-LMP2A (Fig.2A and C). However, H-2D^d^ and H-2K^d^ expression was slightly decreased in three of the four excised tumors when compared with the cell line maintained in vitro (compare Fig.2B and D with Fig.2A and C). The Mean Fluorescence Intensity of H-2D^d^ among the in vitro-cultured Renca-LMP2A cells was 108, whereas among Renca-LMP2A cells obtained from three of the four freshly harvested tumors (on day 21) MFIs were 56, 54, and 70. Similarly, whereas the MFI of H-2K^d^ was 331 for in vitro-cultured Renca-LMP2A, among three of the four tumors, the MFIs were 165, 107, and 163. The remaining fourth tumor did not show a decreased expression of H-2D^d^ (MFI; 148) or H-2K^d^ (MFI 541), however, this was the smallest of the four tumors (with a tumor volume of 50 mm^3^, compared with the other three; 845, 925, and 1012 mm^3^). Taken together these data demonstrate that these Renca tumors may show reduced MHC class I expression as they progress. The finding that there is reduced expression of MHC class I and LMP2A antigen in vivo clearly indicates that Renca-LMP2A tumors have similar phenotypic characteristics to many naturally occurring and experimental solid tumors.

### MHC class I-restricted CTL recognition of Renca-expressed LMP2A in vitro

The observation that Renca-LMP2A tumors grow at a similar rate to LMP2A-negative Renca-VC tumors, suggests that, as with many EBV-associated tumors in patients, there may be inadequate immune recognition of LMP2A epitopes on Renca-LMP2A cells and/or that Renca-LMP2A cells may not stimulate effective LMP2A-specific CTL responses in vivo. To determine whether or not there is indeed immune recognition of Renca-expressed LMP2A epitopes, we first tested their ability to serve as targets in vitro for recognition by LMP2A-specific T cells that were generated in vivo by priming naïve BALB/c mice with a recombinant vaccinia virus vector expressing the LMP2A protein (Vac-LMP2A).

**Figure 3:**
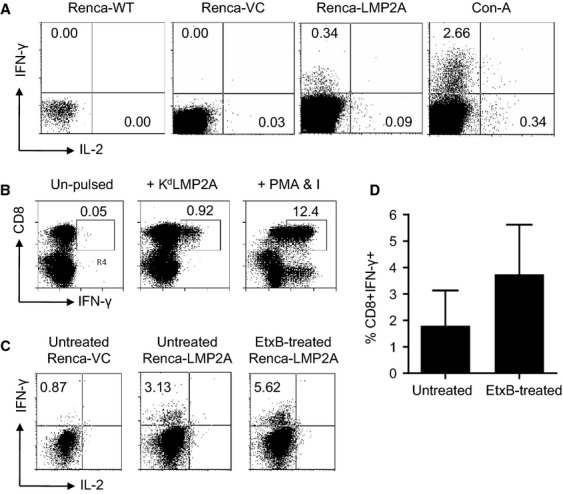
BALB/c mice were immunized with recombinant vaccinia virus Vac-LMP2A at a M.O.I. of 10. Three weeks later splenocytes were harvested and cocultured as follows: (A) with Renca-WT, Renca-VC or Renca-LMP2A as APC or with ConA; panels show flow cytometric analyses of ICS for IFN-*γ* and IL-2 among splenocytes, (B) cocultured with irradiated homologous splenocytes, either unpulsed or pulsed with K^d^LMP2A, or with PMA and ionomycin; panels show flow cytometric analyses of ICC staining of CD8 versus IFN-*γ*; with numbers representing percentage CD8 T cells that are also IFN-*γ*^+^, (C) cocultured with irradiated Renca-VC or with Renca-LMP2A plus or minus EtxB; panels show flow cytometric analyses of ICS for IFN-*γ* and IL-2 among splenocytes, and (D) cocultured with irradiated Renca-LMP2A plus EtxB; bars shows the frequency expressed as a percentage of CD8^+^ IFN-*γ*^+^ double positive cells in the culture after in vitro stimulation for 2 weeks with either untreated Renca-VC and Renca-LMP2A or EtxB-treated Renca-LMP2A APCs.

The results show that up to 3 weeks after immunization with Vac-LMP2A, splenocytes from these mice were not only able to specifically recognize and respond to control Vac-LMP2A-infected target cells in vitro (Fig. S1), but critically, for the purposes of this experimental model, they were able to readily respond to Renca-expressed LMP2A (Fig.3). Evidence of such recognition was shown by the fact that there was positive ICC for both IFN-*γ* and IL-2 after coculture in the presence of irradiated Renca-LMP2A, whereas after coculturing in the presence of Renca-WT or Renca-VC, no IFN-*γ*^+^ or IL-2^+^ splenocytes were detected (Fig.3A). To confirm that there was indeed MHC class I-restricted CD8^+^ CTL recognition of Renca-LMP2A epitopes, splenocytes from Vac-LMP2A-immunized BALB/c mice were cocultured with homologous irradiated splenic APC pulsed with the H-2K^d^-restricted EBV-LMP2A peptide; AAALALLASLIL_305-316_, K^d^LMP2A.

The data show that there was an increase in viable CD8^+^ T cells in the K^d^LMP2A peptide-pulsed cultures compared with the peptide unpulsed cultures (Fig.3B). Evidence of LMP2A-specific CTL effector function was provided by a 20-fold increase in the number IFN-*γ*^+^ CD8 ^+^ cells in the presence of K^d^LMP2A over compared with the unstimulated cultures (Fig.3B). Coculture of splenocytes in the presence of PMA and ionomycin was used as positive control for intracellular cytokine staining. Thus, taken together the data show that there is indeed MHC class I-restricted CTL recognition of Renca-expressed LMP2A; albeit at a very low level.

### Treatment of Renca-LMP2A with EtxB enhances CTL recognition in vitro

Results so far indicate that EtxB is able to enhance CD8^+^ T-cell responses to poorly presented EBV-associated antigens by facilitating their external delivery into the MHC class I cross-presentation pathway, and by improving antigen processing and presentation via the endogenous MHC class I pathway 11–14. Therefore, we wished to test whether or not EtxB could enhance the susceptibility of Renca-LMP2A tumors to LMP2A-specific CTL killing in vivo and control tumor growth. Once again splenocytes from Vac-LMP2A-immunized BALB/c mice were cocultured with Renca-LMP2A cells which had been pretreated with 10 *μ*g/mL of EtxB for 1 hour prior to being used as APC. This resulted in a two-fold increase in the number of IFN-*γ*^+^ CD8^+^ T cells compared with untreated Renca-LMP2A (Fig.3C and D); clearly demonstrating that EtxB treatment of Renca-LMP2A can significantly enhance LMP2A-specific CTL responses, possibly by enhancing antigen processing and presentation in vitro.

### EtxB enhances the CTL recognition of Renca-LMP2A by LMP2A-specific T cells which control tumor growth in vivo

**Figure 4:**
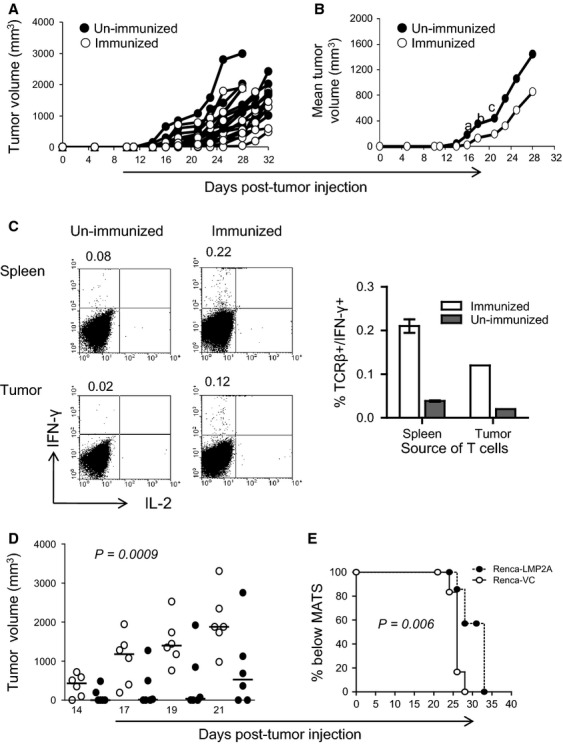
Control of Renca-LMP2A tumor growth in vivo by Vac-LMP2A. On day 0 BALB/c mice were immunized with recombinant Vac-LMP2A (*n* = 10) or unimmunized (*n* = 10) 11 days later, all mice from both groups were injected with Renca-LMP2A, and tumor growth measured up until day 32. Graphs depict (A) individual and (B) mean Renca-LMP2A tumor growth among unimmunized (•), and Vac-LMP2A-immunized mice (○). Two-tailed Mann–Whitney nonparametric test was used to compute the *P*-values for: ^a^ day 14; *P* = 0.007, ^b^ day 16; *P* = 0.004, and ^c^day18; *P* = 0.02. (C) At the end of the tumor-monitoring period, cells were isolated from the spleens and tumors from three mice in each group and used to set up T-cell cultures using irradiated Renca-LMP2A as APCs. After three successive weekly restimulations, cultures were tested for IFN-*γ* and IL-2 production by ICS. Representative FACS plots show percentage of IFN-*γ* versus IL-2 among splenocytes and the bar graph shows the mean ± SEM of IFN-*γ*+ TCR*β*+ cells among cultured splenocytes (spleen, *n* = 3) or TIL (pooled *n* = 3 tumors) from immunized or unimmunized animals. Numbers at the top of the FACS plots indicate the percentage of IFN-*γ*+ cells. D&E Mice immunized with Vac-LMP2A were challenged on day 11 with either Renca-VC (•) or Renca-LMP2A (○) and tumor growth monitored periodically as a surrogate for immune control. (D) Scatter plot showing tumor volumes of individual mice. Horizontal lines indicate the median. One-way ANOVA and nonparametric analysis was used to compute the *P*-value. (E) Top rank analysis of time taken to reach the MATS.

The fact that EtxB significantly enhanced the ability of Renca-LMP2A cells to stimulate effector CTL responses in vitro urged us to test whether or not EtxB could enhance Renca-LMP2A recognition by CTL in vivo; potentially limiting tumor growth. To this end, we first set up a model system in which BALB/c mice were again immunized with Vac-LMP2A, to establish a low level LMP2A-specific CTL response, or left untreated and then injected with Renca-LMP2A and tumor growth was monitored. Although all mice developed tumors, there was a short delay in the onset of tumor growth (2–3 days) among mice immunized with Vac-LMP2A as well as significantly lower tumor volumes between days 11 and 18 indicating low level CTL responses that are generated from immunization with Vac-LMP2A do control tumor growth in the early days (Fig.4A and B). However, during the latter stages, tumor progression among immunized mice occurred at a similar rate as in nonimmunized mice suggesting that there is only a limited potential of these LMP2A-specific T cells to control Renca-LMP2A tumor growth.

This inability to control tumor growth in vivo could be due to limited numbers of LMP2A-specific T cells within the tumor microenvironment or inefficient processing of LMP2A antigens expressed on Renca tumors. Therefore, we first sought to determine whether or not LMP2A-specific T cells could access the tumors. Thus, we compared the frequency of LMP2A-specific T cells within the spleen and tumor-infiltrating lymphocytes (TIL) of Vac-LMP2A-immunized mice. LMP2A-specific TCR*β*^+^ IFN-*γ*^+^ cells were consistently detected in the spleen and within pooled TIL samples (Fig.4C). As expected, the frequency of LMP2A-specific T cells in unimmunized mice was much lower compared to immunized mice; five and sixfold lower in the spleens and tumors, respectively, (Fig.4C). These findings confirmed two important points: First, that potent LMP2A-specific T cells generated following Vac-LMP2A immunization gain access to the tumors but fail to effectively target and control tumor growth. Second, that Renca-LMP2A tumors are restricted in their ability to prime and generate LMP2A-specific T cells in vivo*;* which is consistent with uncontrolled growth observed in Figure1E. Collectively, these data further support the hypothesis that there is an intrinsic defect in antigen processing and presentation of LMP2A when expressed by solid tumors.

However, as immunization with vaccinia virus can lead to potent nonspecific priming of the immune system, it was important to demonstrate that the partial tumor control observed in Vac-LMP2A-immunized mice was not due to a bystander effect of vaccinia-specific T cells, but rather due to specific targeting of tumor-expressed LMP2A by LMP2A-specific CD8^+^ T cells. Therefore, another control experiment was set up in which Vac-LMP2A-immunized mice were challenged with either Renca-LMP2A or Renca-VC tumors. The data show that mice challenged with Renca-VC not only had significantly higher tumor volumes, *P* = 0.0009, (Fig.4D) at all the time points tested, but the time to reach MATS was significantly reduced, *P* = 0.006, (Fig.4E), compared with mice challenged with Renca-LMP2A. The uncontrolled growth of Renca-VC tumors, compared with Renca-LMP2A tumors, in immunized mice confirmed the specific CTL targeting of LMP2A epitopes expressed by the Renca-LMP2A tumors.

**Figure 5:**
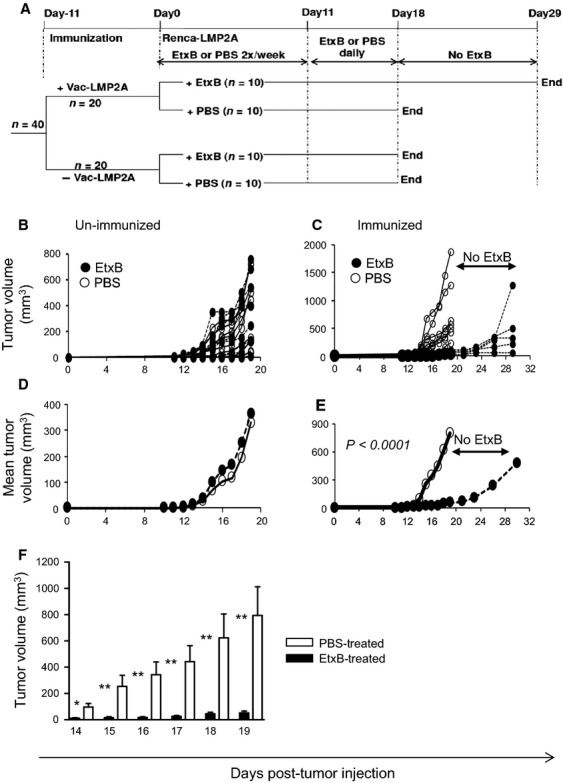
Enhanced immune control of tumor growth by frequent administration of EtxB. (A) Schedule for immunization and EtxB (or PBS) for experiments shown in B–F. (B and D) Unimmunized or (C, E, and F) Vac-LMP2A-immunized mice were injected with Renca-LMP2A tumor cells 11 days later. Mice were then injected with either 50 *μ*g EtxB (•) or PBS (○) two times per week up to day 10, and then daily from day 11 to day 18. Tumor growth was monitored for 18 days, except for the Vac-LMP2A-immunized EtxB-treated mice in which tumors were monitored for a further 10 days without EtxB (as indicated). (B) Tumor volumes for individual mice in the unimmunized group or (C) the immunized group. (D) Mean tumor volumes for the unimmunized or (E) Vac-LMP2A-immunized group of mice. One-way ANOVA (analysis of variance) was used to compute the *P*-value in E. (F) Bar graph showing significant differences in tumor volumes of EtxB-treated (▪) compared to PBS-treated controls (□) on days 14–19. Data is presented as mean + SEM. Two-tailed Mann–Whitney nonparametric test was used to compute the *P*-values; *P ≤ 0.03; **P ≤ 0.003.

The data thus far show that, despite the presence of tumor-infiltrating LMP2A-specific CTL (generated by Vac-LMP2A immunization), the fact that there is slow but progressive growth of Renca-LMP2A tumors clearly suggests that there is only partial control of tumor growth. EtxB has been shown to colocalize with LMP2A in the lipid rafts of EBV-infected LCL where it enhances antigen presentation leading to increased CTL targeting of the LCL 14. We have also demonstrated enhanced targeting of LMP2A in vitro following treatment of Renca-LMP2A with EtxB (Fig.3D and E). We next tested the ability of EtxB to enhance the in vivo antitumor response to Renca-LMP2A. Once again, groups of BALB/c mice were either immunized with Vac-LMP2A to generate an underlying LMP2A-specific CTL response or left unimmunized as controls. Eleven days later, these mice were challenged with either Renca-LMP2A in PBS (PBS controls) or Renca-LMP2A in PBS containing 50 *μ*g of EtxB. The mice were continued on either PBS or EtxB treatment twice a week for the first 10 days. As soon as the tumors became palpable (from day 10 after tumor challenge), EtxB or PBS was administered daily for 8 days, as outlined in Figure5A. EtxB delivery was then stopped on day 18 and the rest of the animals culled; except for the immunized mice treated with EtxB, in which tumor measurements were taken for a further 10 days in the absence of EtxB treatment.

Among the unimmunized mice, there was no difference in the time taken for tumor onset or in the rate of tumor progression; whether or not mice were treated with either EtxB or PBS (Fig.5B and C). However, Vac-LMP2A-immunized mice that were treated with EtxB showed a 5-day delay in tumor onset which was accompanied by significantly lower tumor volumes (*P* < 0.0001) over the entire period of tumor observation (Fig.5D–F). Moreover, in some of the mice in this group tumor progression was halted, whereas in others tumor regression could be observed. Notably, ceasing delivery of EtxB at day 18 led to accelerated tumor growth similar to that observed in Vac-LMP2A-immunized, PBS-treated, control mice; thus highlighting the remarkable potential of EtxB to inhibit tumor progression. These striking observations indicate that EtxB facilitates processing and presentation of Renca-expressed LMP2A thereby resulting in significant tumor control by LMP2A-specific CTL.

### EtxB treatment can achieve long-term control of Renca-LMP2A tumor growth in vivo

**Figure 6:**
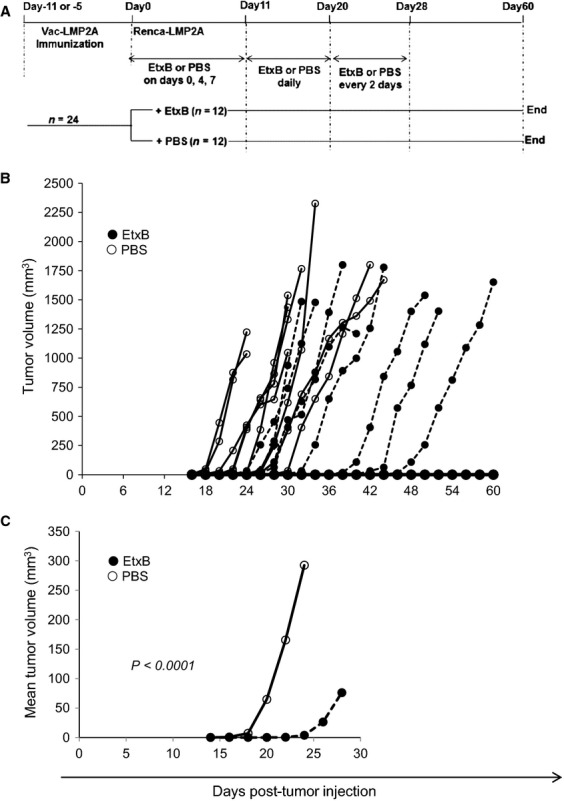
Long-term immune control of tumor growth by EtxB. (A) Schedule for immunization and EtxB (or PBS) for experiments shown in B and C. (B) BALB/c mice were immunized and then injected with Renca-LMP2A 11 days later. EtxB (•) or PBS (○) was administered on days 0, 4, 7, and then daily from days 11–20 followed by once every 2 days until day 28. Tumor growth was monitored for 60 days and is shown as tumor volume for each individual mouse in the group. (C) Mean tumor volumes for each group of mice; One-way ANOVA (analysis of variance) was used to compute the *P*-value.

To determine the longevity of EtxB's impact on delaying tumor onset, new experiments were conducted in which EtxB was administered on days 0, 4, 7, and then daily from days 11 to 20 followed by once every 2 days until day 28 (Fig.6A). Mice were observed and tumor measurements recorded over a 60-day period, provided the tumors did not exceed the MATS. In the PBS-treated group 10 of 12 mice (83%) developed tumors, with only two remaining tumor-free for the entire period (Fig.6B). Tumors in this control group started to grow from day 16 and by day 20 half of the mice (6/12) had palpable tumors, implying that 50% of tumors emerged within the first 20 days. In contrast, all mice in the EtxB-treated group remained free of tumors up to day 20, the first tumor being detected on day 22. Only five of 12 mice (42%) had developed tumors by day 30 compared to 83% in the PBS-treatment group. Three mice in the EtxB group developed tumors much later (on days 36, 38, and 42), whereas four mice remained tumor-free (Fig.6B), demonstrating that EtxB can achieve long-term tumor control. Additionally, the mean tumor volumes in the EtxB-treated group remained significantly lower than those of mice in the PBS-treatment group (*P* < 0.0001); with the maximum difference occurring on day 24 when it was more than 80-fold lower (Fig.6C).

In further studies, the inhibitory effect of EtxB on tumor growth was assessed when tumor inoculation was carried out 5 days after Vac-LMP2A immunization. The rationale for testing this earlier inoculation time point is that the vaccinia response usually peaks around day 7 after immunization and it would presumably have better control of tumor growth, as opposed to day 11 days after Vac-LMP2A immunization when the Vaccinia virus response is starting to decline. However, under these conditions no statistical difference was observed in either tumor incidence, or in the time taken to reach the MATS, between EtxB and PBS-treatment in the two groups (*P* = 0.074 for day 11; *P* = 0.306 for day 5). Taken together our data demonstrate that EtxB can render LMP2A-expressing tumors susceptible to immune control which can significantly delay tumor onset and subsequent growth rate.

## Discussion

The EBV-LMP2A protein is potentially an important tumor antigen that could be utilized in immunotherapeutic and vaccination strategies for EBV-associated tumors as it offers a target for virus-specific CTL that could kill tumor cells 3. In a recent study, it was shown that highly potent LMP2A-specific CTL responses to EBV-associated lymphomas could be generated in patients using transduced DC resulting in a median remission time of over 3 years 5. However, the extent of success among such DC vaccine-based approaches will clearly depend upon the ability of LMP2A-CTL responses to recognize weakly immunogenic tumors that are possibly undergoing immunoediting to escape detection. We have created a novel tumor model in immunocompetent BALB/c mice in which EBV-LMP2A is expressed by solid tumors derived from a well-characterized carcinoma cell line 36. Using this model, we have shown that the nontoxic B subunit of *E. coli* (EtxB) can dramatically limit the growth of LMP2A-expressing solid tumors by rendering them more susceptible to immune control by underlying LMP2A-specific CTL responses.

We have shown that, like many solid tumors, Renca-LMP2A tumors did not stimulate significant antitumor immunity and therefore progressed in an unregulated manner just like the control Renca-VC and the WT Renca tumors. This indicates a lack of significant immune priming by the tumor as well as perhaps the presence of other factors that prevent the formation of an effective antitumor immune response. In support of the former we found that LMP2A-specific T cells were only detected in the in vitro-expanded cultures derived from splenocytes of animals that had been immunized with Vac-LMP2A prior to inoculation of Renca-LMP2A, but not in the unimmunized controls injected with Renca-LMP2A. Nevertheless, there are several reasons which could explain why most tumors are resistant to immune targeting such as (i) the absence of costimulatory molecules, (ii) downregulation of antigen 39 and/or MHC class I molecules 40, (iii) downregulation of pro-apoptotic proteins, secretion of immunosuppressive factors by either the tumor or host cells 41–43, and (iv) the presence of regulatory T cells (Treg) in the tumor microenvironment 44–46. Most importantly, limitations in the processing and presentation of certain tumor-associated antigens such as LMP2A on EBV-associated cancers can adversely affect immune surveillance, hence uncontrolled tumor progression.

We showed that Renca-LMP2A tumors stably express high levels of MHC class I in vitro and in vivo, the slight downregulation that was observed in vivo may result from exposure to certain factors within the tumor microenvironment that may modulate MHC expression 40. Furthermore, loss of tumor-specific antigens does not arise in the case of the Renca-LMP2A tumor model because these tumors expressed LMP2A antigen both in vitro and in vivo. Recognition of LMP2A antigen expressed on Renca-LMP2A tumors, though limited, was demonstrated by partial control of Renca-LMP2A as opposed to the uncontrolled growth of Renca-VC tumors in Vac-LMP2A-immunized mice. It is possible that reduced in vivo expression of LMP2A could have contributed to limiting immune control of tumor growth and eventually causing tumor escape and progression. However, evidence from EtxB experiments where enhanced immune control was achieved despite the documented antigen loss suggests that level of LMP2A expression was not a major limiting factor. In fact these observations indicate that despite LMP2A downregulation, there still remains sufficient antigen to invoke an antitumor response capable of altering tumor growth, but only when efficiently processed and presented to T cells with the help of EtxB.

We have shown that EtxB treatment significantly reduces tumor growth in Vac-LMP2A immunized animals compared to the immunized animals receiving PBS treatment. However, EtxB treatment has no effect on tumor growth in the absence of immunization, possibly due to lack of functional LMP2A-specific T cells in the tumor microenvironment. This therefore calls for further research into the understanding of the modulatory effect of EtxB on solid tumors as it still remains possible that under optimal conditions EtxB could enhance recognition of Renca-expressed LMP2A beyond the threshold that will allow priming of potent LMP2A-specific T cells without the need for prior immunization with vaccinia constructs.

In a few animals the EtxB effect was not observed, possibly due to technical factors such as inconsistent delivery of EtxB to the tumor mass during the early days before tumors became visible and an inability of EtxB to penetrate the tumor mass as the tumors increased in size. This is corroborated with the observation that EtxB-treated tumors begin to progress rapidly when EtxB treatment was terminated, or when administered every 2 days rather than daily, implying that the continuous presence of EtxB is a requirement for the sustained antitumor immune response that prevents tumor progression. As it may be difficult to achieve daily EtxB injections in a clinical setting, it will therefore be necessary to devise possible ways to continuously deliver EtxB.

In full consideration of the profound loss of antigen observed by Renca-LMP2A tumor cells in vivo, and the fact that EtxB has to act on LMP2A to cause redirected trafficking into the MHC class I processing pathway, it is important to note that expression of LMP2A by the tumor cells would be crucial for any EtxB effect. This argument therefore highlights the potential use of EtxB to enhance protective antitumor immune responses even at extremely low levels of antigen expression. Furthermore, since we cannot rule out other mechanisms not tested in this study such as the well-documented massive recruitment and infiltration of Tregs 44 and MDSCs 43 into tumors, one can only speculate that EtxB enhances antitumor immunity despite the immunosuppressive tumor microenvironment. However, this will need to be confirmed in further studies. Although, not directly tested in this study, it is unlikely that the profound EtxB effect was simply due to an additional adjuvant effect rather than enhanced LMP2A trafficking into the MHC class I processing machinery. Suffice to say that any additional adjuvant effect must be minimal due to the fact that there is uncontrolled progression of tumors in unimmunized mice that were also treated with EtxB. In conclusion, we have shown that EtxB has great potential to enhance antigen recognition and generation of antitumor immunity capable of altering the course of tumor growth, irrespective of the tumor-mediated immune escape strategies, and could be harnessed in anticancer vaccine development strategies. Thus the therapeutic use of EtxB could significantly enhance the efficacy of vaccination strategies so as to prolong patient survival much further and to also help to counteract the effects of tumor-mediated immunesuppression, which may cause various different EBV-associated tumors to escape CTL killing.

## Supporting Information

**Figure S1.** Immunization of BALB/c to generate LMP2A-specific CTLs. (A) Monolayers of B2-1 cells were infected with recombinant vaccinia virus Vac-LMP2A or wild type vaccinia virus Vac-WR for 16 h at a multiplicity of infection (MOI) of 10. Confocal immunefluorescence images show LMP2A expression in green and DAPI staining of the nucleus in blue. (B) BALB/c mice were immunized with 2 × 10^5^ p.f.u. of Vac-LMP2A or Vac-WR. Two weeks after immunization, splenocytes were harvested and tested for production of IFN-*γ* and IL-2 after overnight ex vivo restimulation with irradiated B2-1 cells that were: uninfected, Vac-LMP2A-infected, or Vac-WR infected; as APCs. A 6-hour stimulation of uninfected splenocytes with Con-A alone was used as a positive control.
