# Supplementary material for: The B subunit of Escherichia coli enterotoxin helps control the in vivo growth of solid tumors expressing the Epstein–Barr virus latent membrane protein 2A
Source: Cancer Med. 2015 Feb 2;4(3):457–71. doi: 10.1002/cam4.380 (PMC4380971; doi:10.1002/cam4.380)
Supplement: Supplementary file 1 [file cam40004-0457-sd1.pptx]

## Slide 1
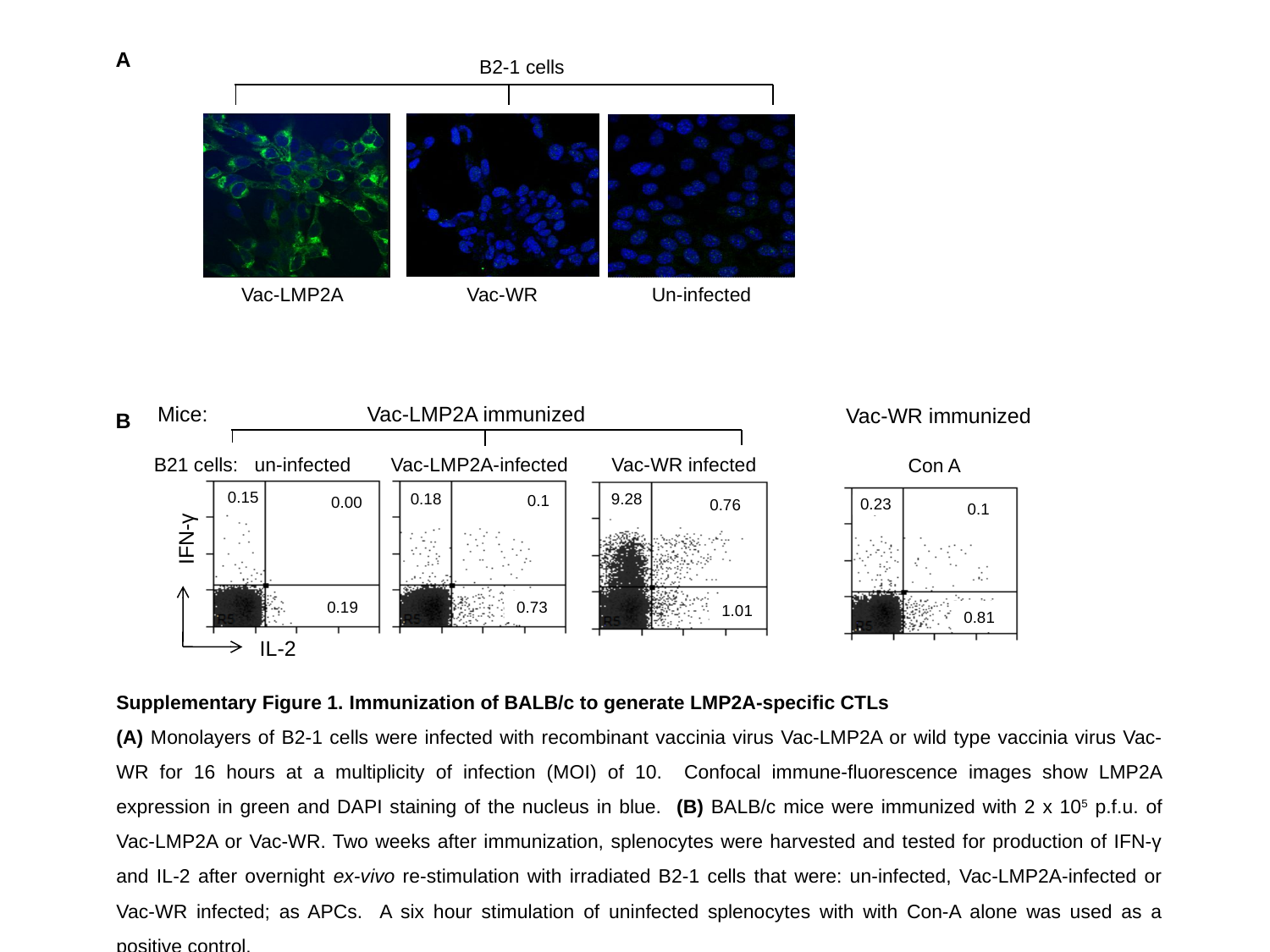

A
B2-1 cells
Vac-LMP2A
Vac-WR
Un-infected
Mice:	 Vac-LMP2A immunized
Vac-WR immunized
B
 B21 cells: un-infected
Vac-LMP2A-infected
Vac-WR infected
Con A
0.15
0.18
9.28
0.1
0.00
0.23
0.76
0.1
IFN-γ
IL-2
0.73
0.19
1.01
0.81
Supplementary Figure 1. Immunization of BALB/c to generate LMP2A-specific CTLs
(A) Monolayers of B2-1 cells were infected with recombinant vaccinia virus Vac-LMP2A or wild type vaccinia virus Vac-WR for 16 hours at a multiplicity of infection (MOI) of 10. Confocal immune-fluorescence images show LMP2A expression in green and DAPI staining of the nucleus in blue. (B) BALB/c mice were immunized with 2 x 10^5^ p.f.u. of Vac-LMP2A or Vac-WR. Two weeks after immunization, splenocytes were harvested and tested for production of IFN-γ and IL-2 after overnight ex-vivo re-stimulation with irradiated B2-1 cells that were: un-infected, Vac-LMP2A-infected or Vac-WR infected; as APCs. A six hour stimulation of uninfected splenocytes with with Con-A alone was used as a positive control.
